# A suppressor locus for MODY3-diabetes

**DOI:** 10.1038/srep33087

**Published:** 2016-09-26

**Authors:** Miguel A. Garcia-Gonzalez, Claire Carette, Alessia Bagattin, Magali Chiral, Munevver Parla Makinistoglu, Serge Garbay, Géraldine Prévost, Cécile Madaras, Yann Hérault, Michel Leibovici, Marco Pontoglio

**Affiliations:** 1 Laboratoire d’ Expression Génique, Développement et Maladies (EGDM), Département Développement, Reproduction et Cancer, INSERM U1016, Institut Cochin, Paris, France; 2 Centre National de la Recherche Scientifique, CNRS UMR7104, Paris, France; 3 Université Paris Descartes, Sorbonne Paris Cité, Paris, France; 4 Institut de Génétique et de Biologie Moléculaire et Cellulaire, Université de Strasbourg, 1 rue Laurent Fries, 67404, Illkirch, France; 5 Centre National de la Recherche Scientifique, UMR7104, Illkirch, France; 6 Institut National de la Santé et de la Recherche Médicale, U964, Illkirch, France

## Abstract

Maturity Onset Diabetes of the Young type 3 (MODY3), linked to mutations in the transcription factor *HNF1A,* is the most prevalent form of monogenic diabetes mellitus. HNF1alpha-deficiency leads to defective insulin secretion via a molecular mechanism that is still not completely understood. Moreover, in MODY3 patients the severity of insulin secretion can be extremely variable even in the same kindred, indicating that modifier genes may control the onset of the disease. With the use of a mouse model for HNF1alpha-deficiency, we show here that specific genetic backgrounds (C3H and CBA) carry a powerful genetic suppressor of diabetes. A genome scan analysis led to the identification of a major suppressor locus on chromosome 3 (*Moda1*). *Moda1* locus contains 11 genes with non-synonymous SNPs that significantly interacts with other loci on chromosomes 4, 11 and 18. Mechanistically, the absence of HNF1alpha in diabetic-prone (sensitive) strains leads to postnatal defective islets growth that is remarkably restored in resistant strains. Our findings are relevant to human genetics since *Moda1* is syntenic with a human locus identified by genome wide association studies of fasting glycemia in patients. Most importantly, our results show that a single genetic locus can completely suppress diabetes in *Hnf1a*-deficiency.

Hepatocyte Nuclear Factor 1 alpha (*HNF1A*) encodes for a transcription factor expressed in liver, kidney, intestine and pancreas. Mutations in this gene lead to Maturity Onset Diabetes of the Young type 3 (MODY3)^1^. This genetic defect represents the most prevalent form of monogenic diabetes^2^. HNF1alpha-deficiency leads to an insulin secretion defect that is characterized by a significant phenotypic variability^3^. Indeed, even in the same kindred, patients carrying the very same mutation may develop diabetes during childhood whereas other members of the family may develop hyperglycemia only after 50 years of age^3^. It has been postulated that this variability may be ascribed to the effect of modifier genes. In support of this hypothesis, a genome scan on different MODY3 families has demonstrated the existence of loci in linkage with the age of onset of the disease^4^. One of the limitations of human genetics approach is represented by the complexity of the interaction between the nature of the mutation and the phenotype^5^. These limitations prevented the identification of the genetic variations responsible for these effects. To circumvent this problem we took advantage of mouse genetics and in particular of a mouse model that recapitulates the main phenotypic traits of MODY3. It has been previously shown that *Hnf1a*^−/−^ mice tend to have smaller Langerhans islets and exhibit a profound defect in glucose-dependent insulin secretion that is comparable to that presented by MODY3 patients^6^,^7^. In the kidney, a specific set of sodium dependent co-transporters including *Slc5a2* is defectively expressed leading to renal Fanconi syndrome characterized by massive glucose, phosphate and amino acid urinary wasting^8^. In a similar way, MODY3 patients suffer from a reduced maximal renal reabsorption capacity for glucose^8^. It has been shown that *Hnf1a*-deficiency leads to a reduced nutrient secretagogue-induced insulin release that is linked to impaired glycolysis^9^ and uncoupling of mitochondrial oxidative phosphorylation^10^ in beta islets. *Hnf1a*-deficiency leads to the significant loss of expression of a large number of genes that are normally expressed in the endocrine pancreas^11^,^12^,^13^. However, the intimate molecular mechanisms responsible for the typical MODY3 insulin secretion defect as well as the reasons for the highly variable expressivity of MODY3 remain poorly understood.

Here we report the drastic effect of the naturally occurring genetic variations in different inbred mouse strains on the diabetic phenotype linked to *Hnf1a*-deficiency. In particular, we identified and characterized a major locus that is able to suppress the diabetes linked to *Hnf1a*-deficiency.

## Results

### The diabetic phenotype of *Hnf1a* ^−/−^ mice is profoundly affected by the genetic background

**Figure 1:**
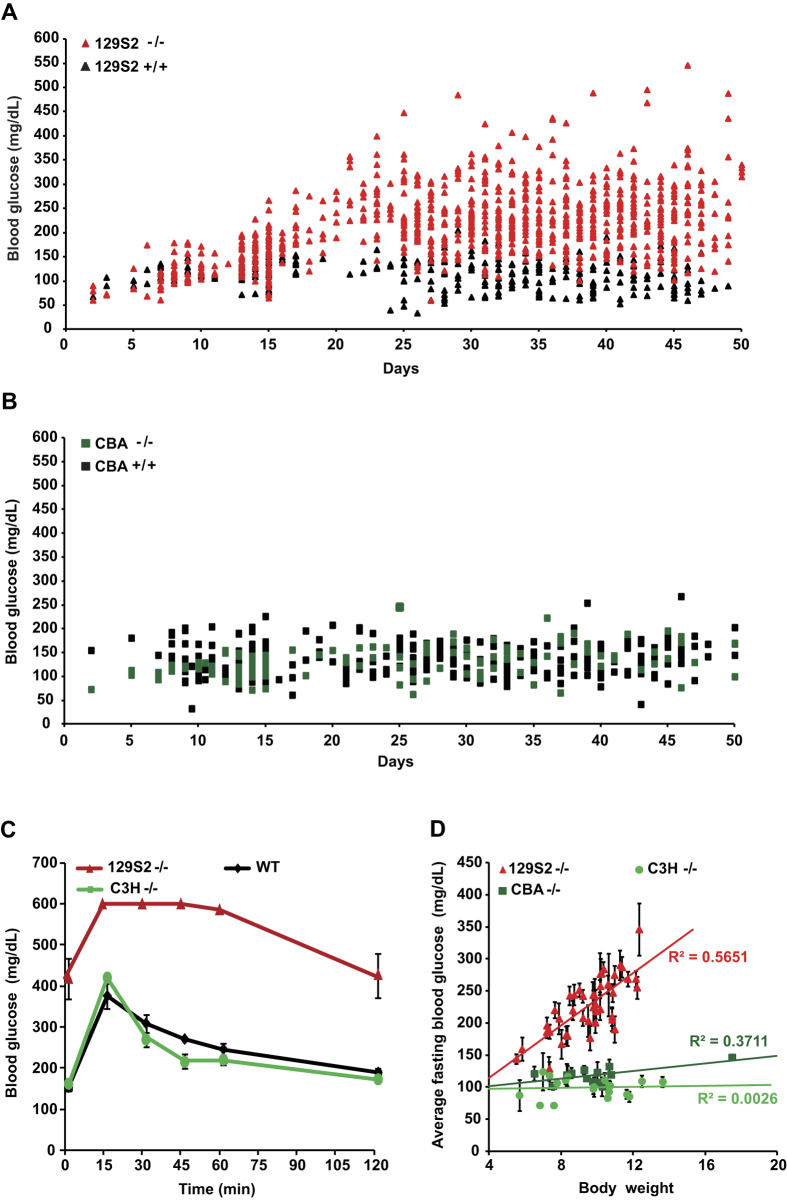
Blood glucose in *Hnf1a* sensitive and resistant mutant strains. (**A**) Blood glucose level in 129S2 *Hnf1a*^−/−^ (129S2^−/−^, red triangles, n = 151) mice and wild-type control (black triangles, n = 54). From weaning onwards, 129S2^−/−^ mice presented significant hyperglycemia compared to control animals (p-value < 0.0001). (**B**) On the contrary, *Hnf1a* mutant CBA strain (dark green squares, n = 41) remained normoglycemic and comparable to CBA wild-type mice (black squares, n = 64). (**C**) Intraperitoneal Glucose Tolerance Test (IPGTT) on sensitive (red, n = 4), resistant (green, n = 4) and wild type (black, n = 3) adult animals demonstrates that the resistant strain is glucose tolerant. Error bars represent SEM. (**D**) Body weight affects the fasting blood glucose level in the sensitive mutant strain (red triangles, n = 43) but has no impact in the two resistant mutant strains CBA (dark green squares, n = 13) and C3H (light green dots, n = 19), respectively. The linear correlation between body weight and fasting glucose level indicated by solid lines is significant only in the sensitive mutant strain (Pearson R^2^ = 0.56). Error bars represent SEM for 3 to 7 fasting blood glucose measurements carried out on mice from 25 to 45 days old (P25 to P45). n = number of animals.

To assess the effect of the genetic background on the diabetic phenotype induced by *Hnf1a*-deficiency, we first carefully characterized the phenotype of *Hnf1a*-null homozygous mice (*Hnf1a*^*tm1Mya/tm1Mya*^ here indicated as *Hnf1a*^−/−^ or more simply mutants) in a pure 129S2/SvPasCrl (129S2) genetic background. Our results showed that mutant mice (n = 151) display normal blood glucose level in the first week after birth. However, these mutants start developing hyperglycemia with values that could rise above 200 mg/dL after weaning at postnatal day 21 (P21) (Fig. 1A). Thereafter, the average difference in blood glucose levels between mutant and wild-type mice was strongly significant (p < 0.0001). To assess the possible impact of the genetic background on the onset and the expressivity of the diabetic phenotype, we introgressed the very same null allele (*Hnf1a*^*t*m1Mya^) into five different genetic backgrounds. Interestingly, our results showed that *Hnf1a* inactivation in a C57BL/6J@Ico (B6) background led to a dramatic perinatal lethality with more than 95% of *Hnf1a*^−/−^ newborns dying in the first 24 hours. In a similar way, the vast majority of BALB/cByJIco (BALB/C) homozygous *Hnf1a*-deficient mice died before weaning. Nevertheless, the few surviving weaned mutant animals in these strains presented hyperglycemia comparable to that observed in the pure 129S2 strain (data not shown). On the contrary, we found that the diabetic phenotype was suppressed in both CBA/JIco (CBA) and C3H/HeNCrI (C3H) *Hnf1a*^−/−^ congenic mice, here indicated as diabetes-resistant (resistant) strains, depicted in green in our diagrams. In fact, as an example, blood glucose levels of CBA mutant mice were overall not significantly different from those of CBA wild-type mice (Fig. 1B). Importantly, intraperitoneal glucose tolerance tests (IPGTTs) demonstrated that adult resistant mutant mice had a glucose tolerance similar to that of control wild-type animals (Fig. 1C). Finally, we showed that the suppression of the phenotype was not due to a possible paradoxically restored expression of *Hnf1a* in resistant strains. In fact, RT-PCR on pancreatic RNA did not show any detectable level of mRNA for this gene in both sensitive and resistant mutant mice (Supplementary Fig. S1).

Similarly to what was previously reported, homozygous null mutant mice displayed a marked growth defect^7^,^14^. However, the extent of this deficit varied considerably even in littermates of the same genetic background. Interestingly, we observed a statistically significant positive linear correlation between growth and hyperglycemia in sensitive strains (see as an example the correlation in 129S2 mutant mice, Fig. 1D). In this respect, we could see that when mutant animals did not reach a body weight of at least 8 g by P25, they tended to remain normoglycemic. Growth deficit could have hindered the development of hyperglycemia in resistant mutant mice. However, our results showed that the average body weight of mutant mice in CBA and C3H strains was comparable to that observed in the sensitive 129S2 background. This indicates that normoglycemia could not be ascribed to growth deficit in these resistant mutant strains (Fig. 1D).

Another important consideration is that *Hnf1a*-deficiency typically leads to an impairment of renal glucose reabsorption^8^. A potentially more severe renal glucose waste could have normalized blood glucose levels of mutants in the resistant strains. However, resistant mutant mice tended to be much less glucosuric compared to sensitive strains, indicating that the blood glucose levels of resistant mutant mice were not corrected by an increased urinary glucose loss (data not shown).

These results indicate that the specific genetic variability of CBA and C3H strains has an intrinsic and genuine ability to suppress the diabetic phenotype elicited by the inactivation of *Hnf1a.*

### F1 intercrosses revealed that the resistance is inherited as a dominant trait

**Figure 2:**
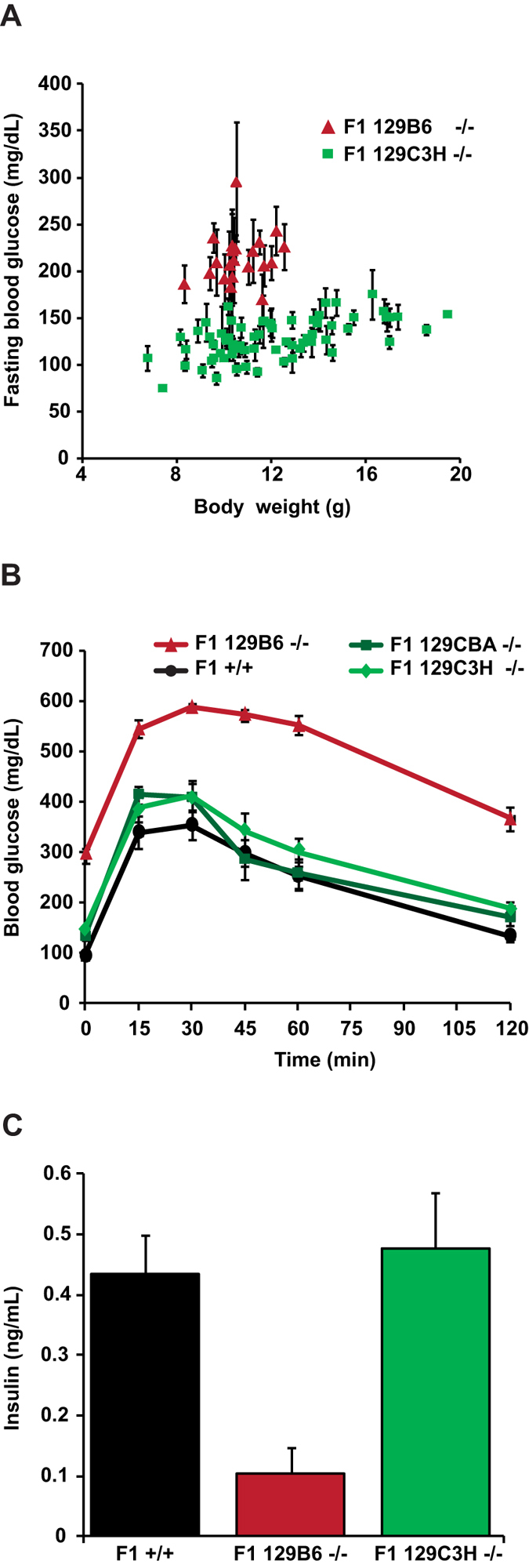
CBA and C3H genetic backgrounds suppress diabetes in F1 *Hnf1a* mutant mice. (**A**) *Hnf1a*^−/−^ mice carrying an F1 129B6 background (red triangles, n = 21) presented hyperglycemia whereas mutant mice carrying F1 129C3H (green squares, n = 69) remained normoglycemic. Fasting blood glucose measurements were carried out on P25-P45 mice. (**B**) Intraperitoneal glucose tolerance test (IPGTT) on F1 sensitive mutant 129B6 strain (red curve, n = 13), F1 resistant mutant strains 129CBA and 129C3H (dark green and light green curves, n = 7 and n = 9, respectively) and F1 129B6 (n = 6) plus 129C3H (n = 6) wild-type mice (black curve). The time course of glucose clearance was comparable in both F1 resistant mutant strains and wild-type mice. (**C**) Plasma insulin levels measured by ELISA at 15 min during IPGTT in F1 wild-type (n = 5), F1 sensitive mutant 129B6 (n = 5) and F1 resistant mutant 129C3H (n = 7). Insulin concentrations were not significantly different between F1 wild-type mice and F1 resistant mutant. n = number of animals. Error bars represent SEM.

In order to characterize the inheritance of the suppression, we monitored the phenotype of F1 *Hnf1a*-null mice issued from a cross between resistant (either CBA or C3H) and sensitive strains (either B6 or 129S2). Our results showed that F1 homozygous mutants obtained from these crosses remained normoglycemic after weaning (Fig. 2A). Conversely, as expected, F1 homozygous mutant animals derived from the cross between sensitive strains presented a clear hyperglycemia (Fig. 2A). Interestingly, resistant F1 mice (from CBA or C3H parents) had glucose tolerance similar to that of wild-type animals (see Fig. 2B as an example). Glucose stimulated insulin secretion (15 min after a glucose intraperitoneal injection) was dramatically impaired in sensitive F1 mutant mice. On the other hand, the insulin secretion of resistant F1 mutant mice was comparable to that of wild-type animals (Fig. 2C). These results indicate that the CBA and C3H genetic backgrounds may contain one or more alleles that behave as modifier loci acting in a dominant way on the suppression of insulin secretion defects.

### Beta cell mass and islet size

**Figure 3:**
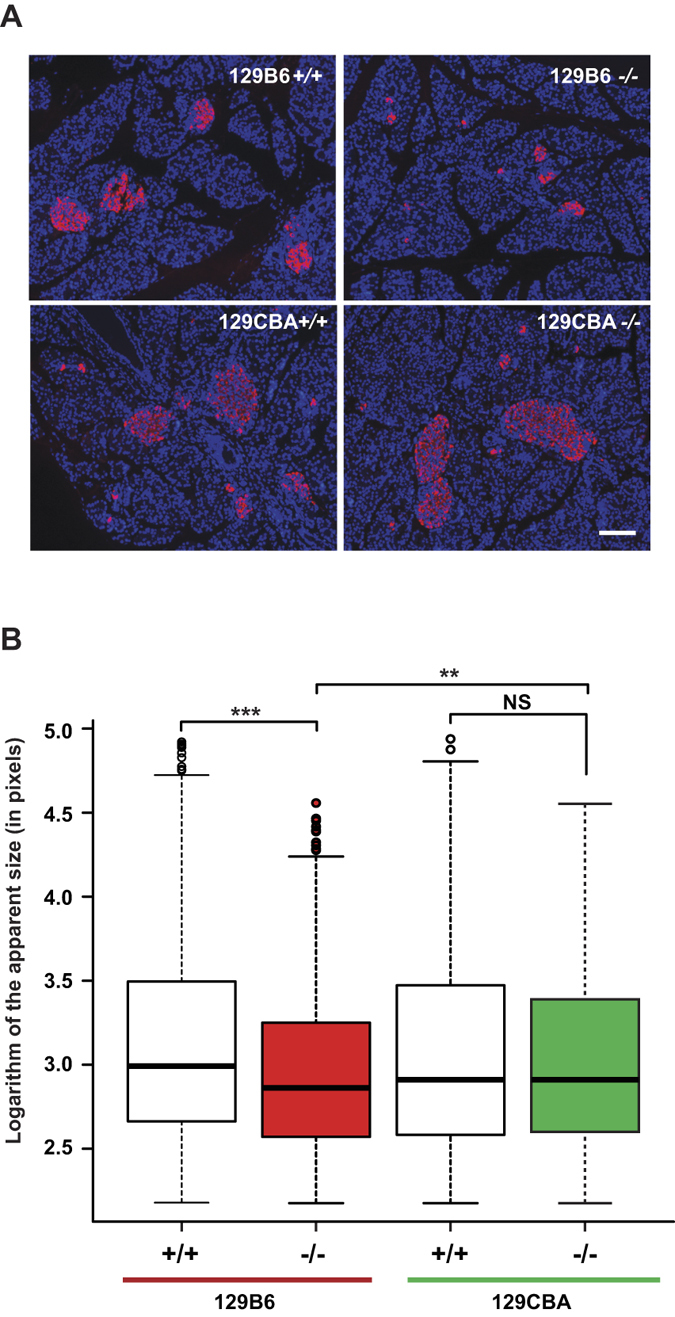
Apparent size of Langherans islets. (**A**) Selected representative views of P15 pancreas histological sections labeled for beta cells (pink staining) and nuclei (blue staining) with insulin antibody and DAPI, respectively. The genotype is indicated on each picture. Scale bar is 100 μm. (**B**) Box plot representation of apparent islet size from each genotype at P15 (3 animals per genotype). The total number of islets analyzed were 1534, 1752, 1433, and 1393 in F1 129B6^+/+^, F1 129B6^−/−^, F1 129CBA^+/+^ and F1 129CBA^−/−^, respectively. Nonparametric Wilcoxon test determined the significant differences between samples (**p-value < 0.005; ***p-value < 0.0001).

We have previously shown that *Hnf1a-*deficiency leads to smaller islet size in mutant compared to wild-type mice^6^. We therefore monitored if the suppression of the diabetic phenotype in resistant mutant mice could be ascribed to a restoration of normal islet development. Our results showed that at P1.5 and P15 both sensitive and resistant F1 mutant strains had a slight decrease in beta cell mass compared to wild-type. However, this difference was not statistically significant (Supplementary Fig. S2). Our morphometric analysis of pancreas histological sections showed that the average apparent islet size of sensitive mutant animals was statistically significantly smaller compared to wild-type at P15. Sensitive mutant mice had therefore more islets but with a smaller size. Remarkably, the islet size of resistant mutant mice was indistinguishable from that of wild-type controls (Fig. 3) indicating that the resistant genetic background had a significant effect in protecting islets from the deleterious effect of *Hnf1a*-deficiency. In addition, we demonstrated that *Hnf1a*-deficiency mice had an increase in beta-cell proliferation and apoptosis that, however, was comparable in both resistant and sensitive mutant strains (Supplementary Fig. S3).

### Genetic analysis of the resistance to develop diabetes

Our results showed that when resistant mice (either CBA or C3H) are crossed with sensitive congenic strains (either 129S2 or B6), the F1 progeny is resistant, indicating that CBA/C3H modifier/suppressor variants act in a dominant way. We hypothesized that these variants might be specific of the CBA/C3H genetic background. In order to identify the chromosomal regions associated with resistance, we carried out a genetic analysis based on a backcross strategy. Since homozygous *Hnf1a*-deficient mice are sterile, mutant mice were obtained via the mating of heterozygous animals. Given the important effect of body weight on the full development of diabetes in the sensitive strain, we decided to increase as much as possible the heterosis (or hybrid vigor) of the animals. To this end, we took advantage of an outbreeding enhancement in the backcross scheme by mating the initial F1 129S2CBA *Hnf1a*^+/−^ mice with a different but nevertheless sensitive *Hnf1a*^+/−^ strain (B6) (Supplementary Fig. S4). In this way, the obtained progeny (referred to as “N2”) received, on the one hand, a complete and uniform complement of B6 chromosomes, and on the other hand, a set of chromosomes represented by either CBA or 129S2 or a recombination between these two strains.

**Figure 4:**
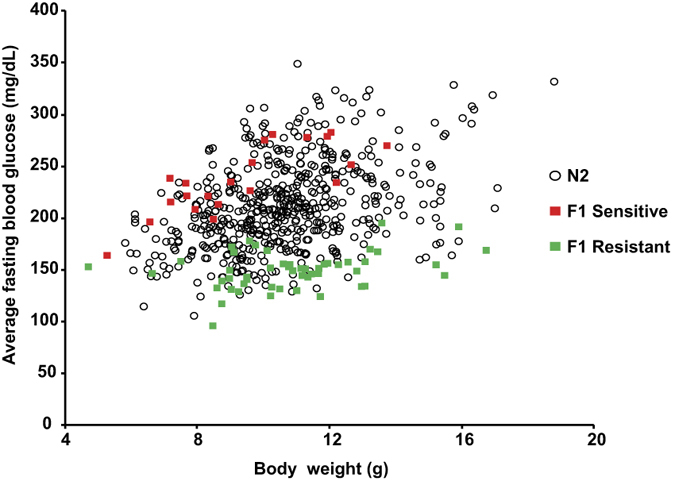
Blood glucose level segregation in N2 *Hnf1a* mutant animals. Blood glucose of F1 sensitive mutant mice (red squares, n = 24), F1 resistant mutant mice (green squares, n = 51) and N2 mutant mice (open circles, n = 532). n = number of animals.

The backcross generated 2100 mice, among which 530 (~1/4) were homozygous for the *Hnf1a*-null mutation. The phenotype of the N2 animals was monitored by measuring blood glucose content, glucosuria and body weight. Phenotypes were assessed between P25 and P45, a temporal window when the resistant and sensitive F1 mutant archetypes displayed an average difference of 4 standard deviations in their average fasting glycemia. Our results showed that the blood glucose levels of the segregating N2 animals were highly variable. Some animals were strictly normoglycemic whereas others developed severe diabetes mellitus (Fig. 4). These data suggested that a relatively small number of loci controlled the suppression of the disease and justified an approach based on a genome scan to identify the loci that could cosegregate with normoglycemia. To this end, we selected a population of N2 *Hnf1a*^−/−^ mice that presented blood glucose levels at the two extremes of the distribution (Fig. 4).

**Figure 5:**
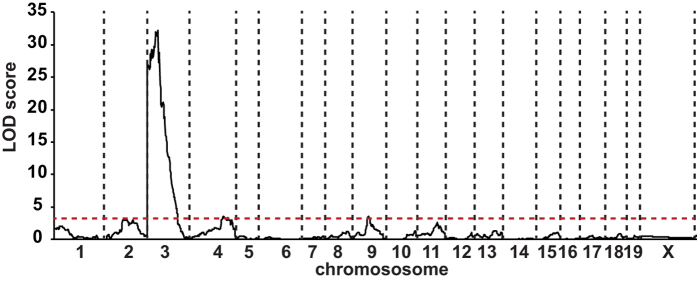
Identification of a major QTL for fasting blood glucose level on chromosome 3. Whole genome multipoint LOD score for the *Moda1* was 32 and defined a critical interval on chromosome 3 of 6.5 Mb (99% confidence interval). The minimum significant threshold value for the LOD score is indicated by a red dashed line.

Since we knew that hyperglycemia develops in a consistent way only in mutant sensitive mice that had reached a minimal body growth, we systematically discarded animals that were below this critical body weight value during the selection of N2 *Hnf1a*^−/−^ normoglycemic mice. In a first round, we selected 28 N2 mice with blood glucose levels comparable with that of the corresponding F1 resistant genetic background. In parallel, we also selected 58 N2 animals that presented blood glucose levels comparable to that of sensitive F1 strain. A genome scan was performed with the Illumina 1,449 SNPs mouse medium density linkage microarray on this first set of 86 N2 mice. This analysis led to the identification of a major Quantitative Trait Locus (QTL) for fasting glycemia on chromosome 3 that we defined as Modifier locus a1 (*Moda1*). The statistical analysis, performed with J/QTL^15^,^16^, identified a 99% confidence interval between 22 and 38 Mb and a LOD score of 21. In order to consolidate this first set of results, we further extended our genetic analysis on an additional population of 55 N2 sensitive and resistant mutant mice with 26 polymorphic markers distributed on *Moda1* and on additional potentially interacting loci. When all the data were pooled, the final LOD score for *Moda1* was increased to 32 (Fig. 5) and the interval was reduced to 27.5–34.0 Mb (99% confidence interval).

**Figure 6:**
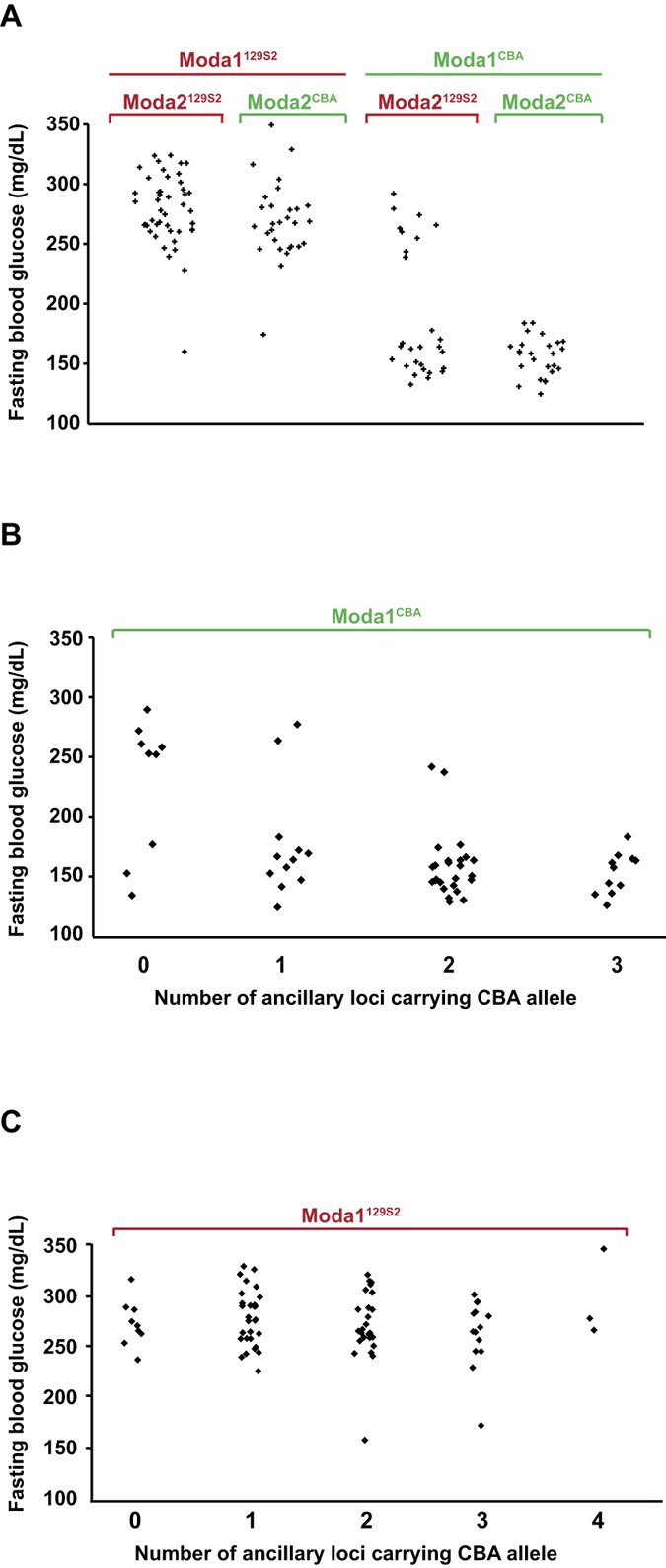
Genetic interaction between the major *Moda1* locus and ancillary loci modulates glucose homeostasis in *Hnf1a*^−/−^ mice. (**A**) Interaction between *Moda1* and *Moda2* loci. When *Moda1* carries the sensitive allele 129S2, almost all mice are hyperglycemic whatever the status of the *Moda2* locus (left part of the chart). Conversely, in animals carrying the resistant CBA allele of *Moda1*, the concomitant presence of *Moda2*^*CBA*^ leads to the suppression of diabetes (right part of the chart). (**B**) Cumulative effect of *Moda3, Moda4* and *Moda5* in mice carrying a *Moda1*^*CBA*^ resistant allele. The progressive decrease of blood glucose level is significantly correlated to the increase, in the genome, of ancillary modifier locus carrying CBA allele (linear regression of 24mg/dL per locus with a p-value < 0.0001). (**C**) The cumulative impact of all ancillary loci has no significant effect on mice that carry a sensitive *Moda1* allele. In all figures each dot represents a mouse. For clarity, the complementary B6 allele was not indicated.

The segregation of *Moda1* explained 65% of the variance of fasting glycemia and had an average effect of 100 mg/dL on blood glucose level. Interestingly, more than 95% of the normoglycemic N2 mice inherited *Moda1* from the resistant CBA strain (*Moda1*^*CBA*^) and conversely, around 90% of hyperglycemic animals inherited the sensitive 129S2 allele (*Moda1*^*129*^) (Supplementary Fig. S5). However, only 83% of the mice that inherited the suppressor locus were actually normoglycemic indicating that the CBA allele of *Moda1* was not sufficient to completely suppress diabetes. In order to identify the possible additional loci that interacted with *Moda1* to suppress diabetes, we screened the combinatorial distribution of alleles in the whole population of N2 mice that we genotyped. Our results showed that 4 additional loci interacted in a statistically significant way (Fisher’s exact test) with *Moda1* in the segregation of fasting blood glucose levels. These ancillary loci, called *Moda2, Moda3, Moda4* and *Moda5*, were centered on chr4:152 Mb, chr11:35 Mb, chr11:112 Mb and chr18:84 Mb (GRCm38/mm10), respectively. Interestingly, the most significant, *Moda2*, in combination with *Moda1*, completely suppressed diabetes (Fig. 6A). On the other hand, the global number of the other resistant ancillary loci (from *Moda3* to *Moda5*) had a concomitant significant effect on blood glucose level of mice that carried the resistant version of *Moda1* (Fig. 6B). Conversely, the level of glucose in N2 animals that inherited the sensitive allele of *Moda1* was not significantly decreased by the presence of resistant ancillary loci (Fig. 6C). These results indicate that the CBA allele of *Moda1* is necessary but not sufficient to completely suppress diabetes.

In order to further validate the suppression of diabetes induced by the presence of *Moda1* locus we took advantage of a consomic strain C3H/HeJ-Chr 3^C57BL/6J^/J. This mouse strain carries an entire chromosome 3 from the B6 strain while the rest of the genome is C3H. We first crossed this strain to introduce the null mutation for *Hnf1a* at the heterozygous state and then we intercrossed to select congenic recombinants with breakpoints encompassing the *Moda1* locus (see Methods and Supplementary Fig. S6). Interestingly, these recombinant mouse strains, when crossed with 129 heterozygous mice to get an F1, presented with a fasting hyperglycemia comparable to that of sensitive F1 mouse strains. In addition, IPGTT elicited an average area under the curve that was very significantly increased, compared to F1 resistant strain (Supplementary Fig. S7). All these results demonstrated that the interval identified by the genetic analysis is actually responsible for the suppression of the development of diabetes.

### The *Moda1* locus

**Figure 7:**
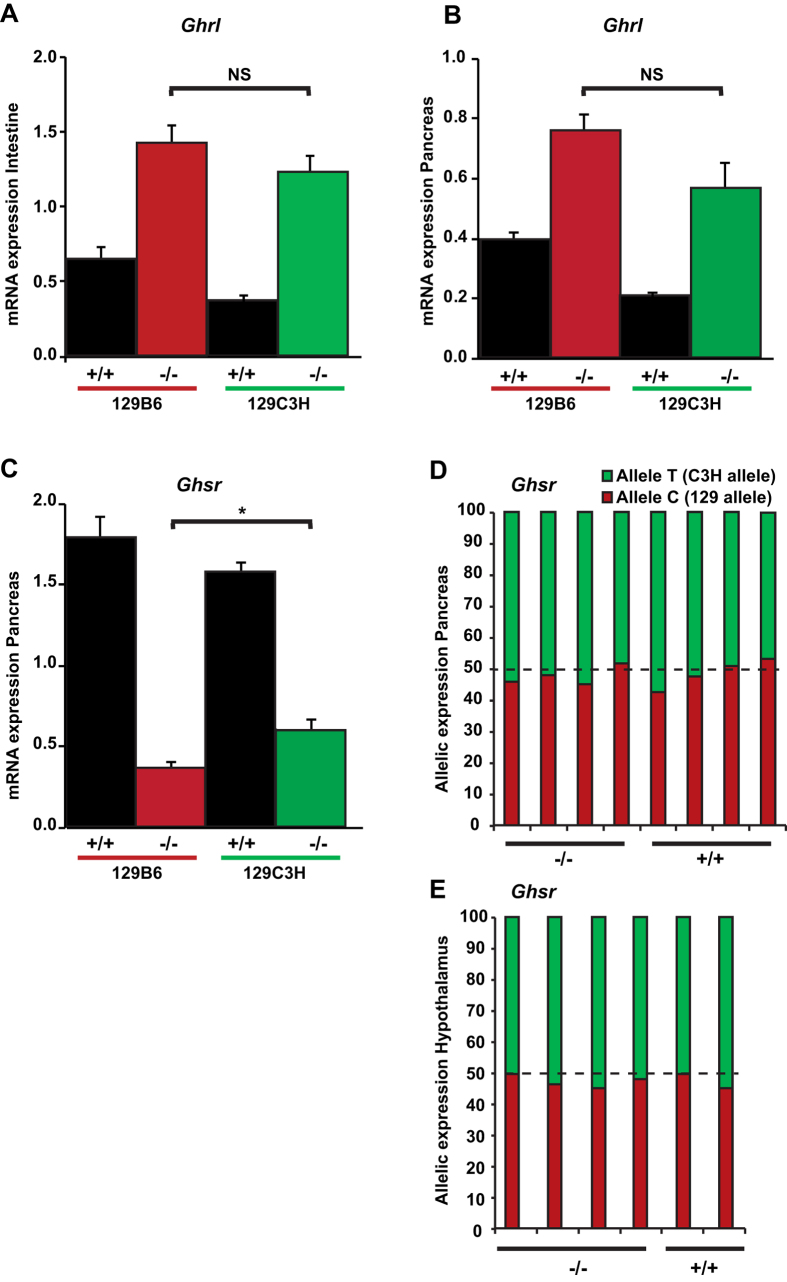
Expression of Ghrelin and Ghrelin receptor genes. *Ghrelin (Ghrl*) and *Ghrelin receptor (Ghsr*) levels were determined by RT-qPCR in F1 wild-type 129B6 and 129C3H mice (n = 4 and n = 4, respectively) and F1 *Hnf1a*^−/−^ 129B6 and 129C3H mice (n = 5 and n = 6, respectively). In (**A**) adult jejunum and (**B**) P6 pancreas, an increase in *Ghrl* transcripts is observed in both mutant strains compared to wild-type mice but no significant differences in expression were observed between the mutant strains. (**C**) By contrast, in P6 pancreas, *Ghsr* transcripts were decreased in mutant strains compared to the wild type counterparts. This decrease was statistically significantly higher in sensitive mutant compared resistant mutant strains (*p < 0.05). NS = non-significant. Allelic expression (pyrosequencing) of the *Ghsr* gene is presented in F1 129C3H mice in (**D**) pancreas, and (**E**) hypothalamus. The results are represented as a percentage of expression from each allele. n = number of animals.

In order to try to understand the nature of the genetic variability responsible for the suppression of the diabetic phenotype elicited by the ablation of *Hnf1a*, we inspected the genes that are located in the *Moda1* critical region. An interesting candidate suppressor gene is represented by the ghrelin receptor (*Ghsr*) that is very close to the *Moda1* locus. Recently, it has been demonstrated that the ligand of this receptor, the ghrelin hormone, is expressed at much higher levels in the jejunum of *Hnf1a*-deficient mice. In this respect, it has been shown that the inhibition of ghrelin receptor activity can restore a normal glucose homeostasis in *Hnf1a*-deficient mice^17^. Our sensitive and resistant strains do not carry coding variants for either ghrelin (*Ghrl*) or ghrelin receptor proteins. However, we could not rule out that a differential expression of *Ghrl* and/or *Ghsr* between these two strains could account for the suppression of diabetes observed in resistant mutant mice. To this end we first monitored the expression of *Ghrl*, whose gene is located on a different chromosomal region that is not genetically associated to resistance. In agreement with what was previously reported^17^, our results showed that this gene is significantly overexpressed in the intestine of both sensitive and resistant mutant mice (Fig. 7A). In addition, we showed that this gene was also overexpressed in pancreas (Fig. 7B). However, the extent of this overexpression was not significantly different between resistant and sensitive mutant mice. We then monitored the expression of the receptor (*Ghsr)* in pancreas and showed that mutant mice had a significant downregulation of the gene compared to wild-type mice (Fig. 7C). Paradoxically, the expression of *Ghsr* in resistant mutant mice was statistically significantly higher compared to that of sensitive mutants. However, given the effect of the ghrelin receptor antagonist on the suppression of diabetes, the increase in *Ghsr* expression in resistant compared to sensitive mutant mice could not account for the resistance in CBA/C3H strains. More importantly, our results obtained with pyrosequencing showed that the transcripts of this gene were equally transcribed from both (resistant and sensitive) alleles both in pancreas and hypothalamus, the two organs where this receptor is known to play a significant role (Fig. 7D,E, respectively). All these results indicate that the *Ghsr* gene does not seem to contribute to the suppression of diabetes in CBA/C3H mice.

**Figure 8:**
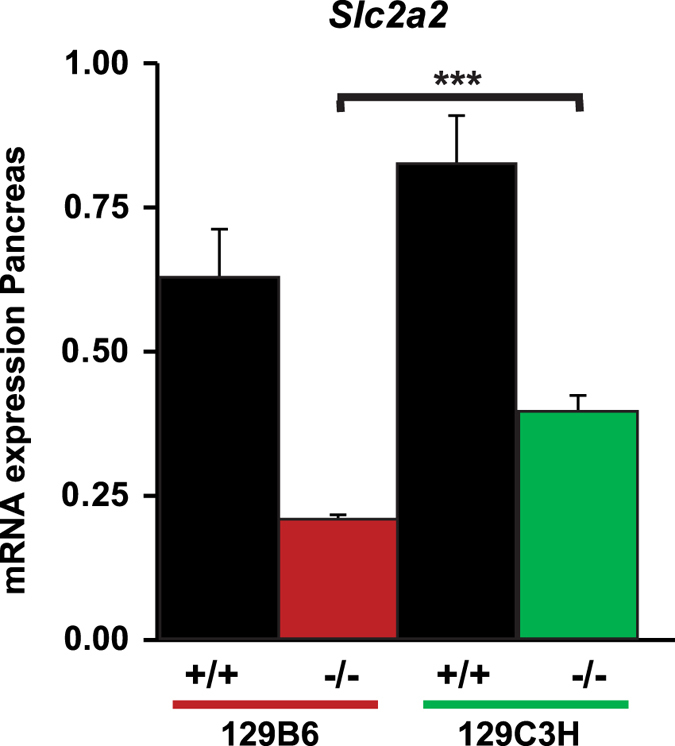
*Slc2a2* expression in sensitive and resistant mutant strains. *Slc2a2* expression was assessed by RT-qPCR in wild-type 129B6 and 129C3H mice, and in *Hnf1a*^−/−^ 129B6 and 129C3H mice in the pancreas at P6 (n = 5 for each genotype). A decrease of *Slc2a2* transcripts is observed in sensitive and resistant mutant mice compared to wild-type mice. Resistant mutant mice express statistically significant higher levels compared to sensitive mutant mice (***p-value < 0.005). n = number of animals.

Interestingly, the peak of significance of *Moda1* was centered on *Slc2a2*, a gene whose specific SNPs have been found to be strongly associated with fasting blood glucose levels in a number of genome wide association studies (GWAS) in patients^18^,^19^. This gene has been shown to play an important role in glucose sensing and insulin secretion of beta cells. Interestingly, *Slc2a2* has also been shown to be a direct transcriptional target of HNF1alpha^20^. The resistant and sensitive alleles for this gene did not differ in any respect in the primary sequence of GLUT2, the protein product of *Slc2a2*. Consistently, our results showed that mutant mice from either sensitive or resistant strains had a substantial reduction in the mRNA expression of this gene compared to wild-type mice. In addition, resistant mutant mice had a modest but statistically significant increase of mRNA expression compared to sensitive mutant mice (Fig. 8).

The residual expression of *Slc2a2* in sensitive mutant mice still remained above 20% of that of control animals. This level of expression was previously demonstrated to be sufficient to maintain glucose homeostasis in mice^21^. In line with this consideration, GLUT2 may not necessarily be the limiting factor leading to diabetes in sensitive mutant mice. Therefore, it is unlikely to imagine that *Slc2a2* is the diabetes suppressor.

In order to get more insights on the nature of the suppressor, we reconsidered the list of genes in the (*Moda1*) critical region by inspecting the genetic variants identified by next generation sequencing^22^. Interestingly, the comparison of the C3H and CBA strains in the *Moda1* locus showed that these strains share the same ancestral DNA since the frequency of SNPs between these two strains was extremely small (1 SNP every 75 Kb) and mostly located in the intergenic regions of *Moda1*. Finally, the analysis of the genetic variations in *Moda1* showed that only 11 genes presented non-synonymous SNP changes between resistant CBA/C3H and sensitive 129S2 and B6 strains (Supplementary Table S1). However, none of the candidate genes identified within the locus has been clearly directly involved in pathways whose defect might lead to diabetes.

## Discussion

In this study, we have shown that the genetic background of different inbred mouse strains has a dramatic impact on the diabetes induced by the deficiency of the transcription factor *Hnf1a*. Indeed, we found that the typical severe diabetic phenotype that is observed in sensitive strains (e.g. 129S2) is completely suppressed by the genetic variation present in CBA and C3H strains. When introgressed in these two strains, the homozygous inactivation of *Hnf1a* no longer elicits any diabetic phenotype, mice are normoglycemic after weaning and notably, have normal glucose tolerance. One important consideration is that the current study has disclosed a novel aspect on the mechanisms that lead to hyperglycemia in HNF1alpha deficiency. The currently accepted model of MODY3 beta-cell dysfunction is believed to be related to a profound alteration of global gene expression in beta cells upon the ablation of HNF1alpha function. In fact, it has been shown that an impressively large number of direct and indirect target genes are differentially expressed in the islets of Langerhans of *Hnf1a*^−/−^ mice^13^,^23^. The novelty of our results stem from the fact that we demonstrate that the naturally occurring genetic variation in a single major locus is sufficient to suppress the series of events that lead to this loss of identity and insulin secretion function.

Our results showed that sensitive and resistant mutant mice had similar beta cell mass. However, when compared to sensitive mutant mice, resistant mutant mice had larger beta islets that were comparable to what is observed in wild-type animals. Islet size has been demonstrated to potentiate the physiological properties of beta cells^24^. Consistently, we observed that islet size in resistant mutant mice correlates with the ability to secrete insulin. Therefore, it is likely that morphologically preserved islet-size in resistant mutant mice may account for the restoration of the insulin secretion. Interestingly, no difference of cell proliferation or apoptosis seems to account for larger beta islets in the resistant mice. On the other hand, we observed that the preservation of islet growth, leading to larger Langerhans islets in resistant mutant mice, seems to occur during postnatal life since at birth mutant pups do not show any significant difference (data not shown). Hence, we propose that a defective aggregation process of beta cells after birth could explain smaller islet size in sensitive mutant mice.

The molecular mechanism leading to insulin secretion defect in HNF1A deficiency is not really completely elucidated. It is known that this altered gene expression that takes place in Langerhans islets in *Hnf1a* deficiency is associated to a defective glycolytic flux^9^ and uncoupling of mitochondrial oxidative phosphorylation^10^. It is worth noting that these two functional metabolic steps are downstream to Slc2a2/GLUT2. In addition, it has been demonstrated that the residual expression level of *Slc2a2* gene, which we have observed in sensitive mutant mice, is still compatible with a normal insulin secretion^21^. This suggests that the reduced expression of *Slc2a2* may not necessarily be the limiting step that leads to diabetes in *Hnf1a* deficiency.

Our results showed that resistant mutant mice suppressed diabetes in spite of the overexpression of *Ghrl*, a hormone whose antagonist had been shown to be able to normalize insulin secretion and hyperglycemia in *Hnf1a* deficiency. This suggests that in the resistant genetic background, the effect of the overexpression of *Ghrl* is not sufficient to elicit insulin secretion defects. Interestingly, the ghrelin receptor gene (*Ghsr*), located near *Moda1* does not contain any non-synonymous SNP and its expression is decreased at the same extent both in sensitive and resistant mutants. More importantly, our results showed that in F1 resistant 129C3H mutant mice, the *Ghsr* gene is equally expressed from both resistant and sensitive alleles. Therefore, the resistant phenotype cannot be ascribed to the *Ghsr* gene itself although we cannot rule out that post-translational modifications of the ghrelin receptor protein linked to the action of other genes may account for a potentially altered receptor activity.

The interest and the novelty of our study stems from the fact that we demonstrated that the mechanisms leading to MODY3 diabetes can be overcome by a single genetic locus even in the absence of the expression of HNF1alpha. The corollary of this consideration is that the events that lead to the development of diabetes are not necessarily unconditionally triggered by the absence of *Hnf1a*. Together, all these considerations indicate that the pathogenic mechanisms of MODY3 are probably more subtle than what was initially thought. *Moda1* locus must contain gene variant(s) whose effect (in CBA or C3H) is able to prevent the disruption of a subtle mechanism, which, in the absence of HNF1alpha, in sensitive strains, triggers the defective growth and/or aggregation of islets. This could generate a situation where beta-cells progressively lose their capacity to secrete insulin in response to glucose. In this respect, we cannot rule out the presence of non-coding mutations that might affect the expression of genes or modify the biological properties of non-coding RNAs. The clarification of the nature of the modifier gene(s) responsible for the suppression will require further investigations. These efforts could shed a novel light for better understanding of the mechanism that leads to diabetes caused by *Hnf1a*-deficiency. In the last years several genome wide association studies have indicated that genes expressed in beta cells, and potentially involved in insulin secretion, might play an important role on the onset of type 2 diabetes mellitus. In this respect, a better comprehension of the gene networks that interact with HNF1A/MODY3 could be extremely useful in dissecting the molecular aspects underlying the variability of insulin secretion defects in type 2 diabetes.

## Methods

### Animal handling and breeding

All experiments with animals were approved by the local Committee (CEEA34.EF.083.12) and were carried out according to our institution guidelines and EU legislation. Animals were housed in cages at room temperature with a 12 h light/dark cycle. Mice were weaned at P21 and given access to water and rodent chow that was removed 16 h before the experiments in fasting conditions. The *Hnf1a*^*t*m1Mya^ mutation (here called *Hnf1a*^−/−^) has been introgressed by repeated backcrossing over 10 generations in the following mouse inbred strains: 129S2/SvPasCrl (129S2), C57BL/6J@Ico (B6), BALB/cByJIco (BALB/C), CBA/JIco (CBA) and C3H/HeNCrI (C3H). All mice were obtained from Charles River Laboratories. Since homozygous *Hnf1a-*deficient mice are sterile, heterozygous animals were mated in order to obtain homozygous mutant mice. The breeding strategy to generate N2 mice is depicted in Supplementary Figure S4 and described in detail in the Results section. The consomic strain C3H/HeJ-Chr 3^C57BL/6J^/J was obtained from the Jackson laboratory (strain number 006359) and used to generate congenic strains that were recombined in the vicinity of the *Moda1* locus. The detailed breeding strategy is presented in the Supplementary Figure S6. The recombinant animals were then mated with 129 *Hnf1a*^*+/−*^ mice and *Hnf1a*^−/−^ progeny was challenged for glucose by IPGTT (see below) to determine their sensitive or resistant phenotype to diabetes.

### Genotyping

Genomic DNA was isolated from mouse tail tips and subjected to PCR using GoTaq G2 polymerase (Promega). Primer sequences are listed in Supplementary Table S2.

### RNA isolation and reverse transcription

After dissection in PBS, mouse tissues were snap-frozen in liquid nitrogen and stored at −80 °C. RNAs were extracted from frozen samples with TRIzol as described by the manufacturer (Life Technologies). Since the pancreas is highly enriched in RNases, the frozen tissue was ground to powder in liquid nitrogen before being resuspended in TRIzol, and subjected to three rounds of TRIzol extraction. The quality of the isolated RNA was assessed on a Bioanalyzer 2100 (Agilent). For all samples, reverse transcription was performed on 2 μg of RNA with the High-capacity cDNA reverse transcription kit (Applied Biosystems), with or without reverse transcriptase.

### qPCR and Pyrosequencing

Diluted cDNAs were amplified using the GoTaq^®^qPCR Master Mix (Promega) on a LightCycler Mx3005 qPCR system (Agilent). In order to check the allelic expression of *Ghsr*, we identified a 3′ UTR SNP in the *Ghsr* locus of 129S2 and C3H strains that is a single nucleotide variant using the Sanger Mouse Genome Project SNP/Indel query. Amplification and sequencing primers were designed using the PyroMark Assay Design Software (Qiagen). cDNAs from P6 pancreas and adult hippocampus of either wild-type or *Hnf1a*^−/−^ 129C3H mice were amplified with PCR primers corresponding to a region covering the SNP to be analyzed. The biotinylated PCR products obtained were purified with Streptavidin sepharose high performance beads (GE Healthcare 17-5113-01). The products were annealed with the sequencing primer and subjected to pyrosequencing using PyroMark Q24 system (Qiagen). Primers used are listed in Supplementary Table S2.

### *In vivo* studies

All experiments were performed on conscious mice. Blood glucose level was measured on tail blood with Accu-Chek Performa glucose meter (Roche) in fasting and non-fasting conditions. Intraperitoneal Glucose Tolerance Tests (IPGTTs) consisted injection of 2 g D-glucose/kg in fasting adult male mice. Blood glucose was then measured at 0, 15, 30, 45, 60 and 120 min. During IPGTTs, 100 μL of blood was collected from the tail vein at 0 and 15 min, and the insulin content was determined in the recovered plasma using the Mouse Ultrasensitive Insulin ELISA kit (Alpco). Glucosuria was monitored with Diabur-test 5000 strips (Roche).

### Genome scan for Quantitative Trait Loci (QTL), candidate gene detection and genetic interaction

The genome scan was performed on genomic DNA extracted from spleen or liver from the 28 most resistant (that had reached a minimal body growth of at least 8 g at P25) and the 58 most sensitive N2 *Hnf1a*^−/−^ mice using a panel of 1449 polymorphic SNPs available in the Illumina-GoldenGate Mouse Linkage Panel (OPA GS0006826). 707 SNPs are different between 129S2 and CBA strains allowing to discriminate allelic contribution of each strain. SNP genotyping was carried out on the Integragen Illumina microarray platform (Evry, France). A second set of 22 resistant and 33 sensitive N2 *Hnf1a*^−/−^ animals were manually genotyped for 26 polymorphic markers (see Supplementary Table S2). In the QTL region, modifier candidate genes were searched based on bioinformatic detection of non-synonymous SNPs between sensitive and resistant strains with publicly available SNP data from the last version of the Mouse Genomes Project (https://www.sanger.ac.uk/sanger/Mouse_SnpViewer/rel-1303)^22^.

### Beta cell mass determination, apoptosis and proliferation analysis

The beta cell mass was determined on F1 wild-type 129B6 and CBAB6, and F1 *Hnf1a*^−/−^ 129B6 and CBAB6. Three males of each genotype were processed at P1.5 and P15, respectively. Dissected pancreases were weighted and fixed in 4% paraformaldehyde overnight and then processed for paraffin sections. Pancreases were oriented such that the sections were cut along the head-tail axis. Paraffin sections of 5 μm thickness were serially collected on slides. Sections were treated overnight at 4 °C with a guinea pig insulin antibody (DAKO), and for 1 h at room temperature with an appropriate secondary antibody (Alexa Fluor goat anti-guinea pig 594, Life Technologies). After staining with DAPI (Life Technologies) and mounting with Fluoromount G (Interchim), each stained pancreas section was completely imaged to reconstitute a unique image of the whole pancreas section considered. Each reconstituted image was analyzed with ImageJ software (http://imagej.nih.gov/ij/) to quantify beta cell area and total tissue area (script for ImageJ developed by Camille Lebugle, Cellular Imaging Platform, Cochin Institute, Paris, France). Beta cell mass was estimated by the following formula: beta cell mass = (beta cell area/total tissue area) x pancreas weight^25^. Islet area data of all mice were extracted and analyzed for the distribution of islets sizes with R software. Results are expressed as mean ± SD. Apoptosis of beta cells was determined on pancreatic sections using the *in situ* Cell Death detection kit according to the manufacturer’s instructions (Roche). The proliferative index was assessed with a rabbit anti-Ki-67 antibody (Novocastra), with overnight incubation at 4 °C. An Alexa Fluor donkey anti-rabbit 488 was then applied on sections for 1 h at room temperature. Beta cells were identified by co-staining with the insulin antibody as described above. Each slide was scanned using fluorescence microscope (Axiovert 200 M, Zeiss) and Metamorph software. Quantitative data were obtained manually using the cell counter plugin in ImageJ software (http://rsb.info.nih.gov/ij/plugins/cell-counter.html). All data are means ± SD.

### Statistical analysis

Values are reported as a mean ± SE, unless otherwise noted. Statistics were calculated using the two-way, two-tailed Student t test or the nonparametric Wilcoxon test. For beta cell mass ANOVA test was used, and genetic interactions were validated using the Fischer’s exact test. Differences were considered significant with a p-value < 0.05.

## Additional Information

**How to cite this article**: Garcia-Gonzalez, M. A. *et al*. A suppressor locus for MODY3-diabetes. *Sci. Rep.* **6**, 33087; doi: 10.1038/srep33087 (2016).

## Supplementary Material

Supplementary Information
