# Erratum: A suppressor locus for MODY3-diabetes

**DOI:** 10.1038/srep35697

**Published:** 2016-10-21

**Authors:** Miguel A. Garcia-Gonzalez, Claire Carette, Alessia Bagattin, Magali Chiral, Munevver Parla Makinistoglu, Serge Garbay, Géraldine Prévost, Cécile Madaras, Yann Hérault, Michel Leibovici, Marco Pontoglio

Scientific Reports 6: Article number: 3308710.1038/srep33087; published online: 09 26 2016; updated: 10 21 2016

The original version of this Article contained a typographical error in Affiliation 2. The affiliation:

Centre National de la Recherche Scientifique, CNRS UMR7104, Paris, France.

Now reads:

Centre National de la Recherche Scientifique, CNRS UMR8104, Paris, France.

This has been corrected in the HTML and PDF versions of this Article.

