# Supplementary material for: A suppressor locus for MODY3-diabetes
Source: Sci Rep. 2016 Sep 26;6:33087. doi: 10.1038/srep33087 (PMC5036084; doi:10.1038/srep33087)
Supplement: Supplementary Information [file srep33087-s1.pdf]

A suppressor locus for MODY3-diabetes

Miguel A. Garcia-Gonzalez[1,2] , Claire Carette[1,3], Alessia Bagattin[1], Magali Chiral[1], Munevver Parla Makinistoglu[1], Serge Garbay[1], Géraldine Prévost[4], Cécile Madaras[1], Yann Hérault[4], Michel Leibovici[1,5] and Marco Pontoglio[1,5*]

[1]Expression Génique, Développement et Maladies Laboratory (EGDM), Département Développement, Reproduction et Cancer, INSERM U1016, CNRS UMR8104, Université Paris Descartes, Institut Cochin, Paris, France.

[2]Present address: Laboratorio de Nefrología, Complexo Hospitalario de Santiago de Compostela (CHUS), Instituto de Investigación Sanitaria (IDIS), Santiago de Compostela, Spain.

[3]Present address: Département de Nutrition, Hôpital Ambroise Paré, Assistance Publique-Hôpitaux de Paris (APHP), Boulogne-Billancourt, France.

[4]Institut de Génétique et de Biologie Moléculaire et Cellulaire (IGBMC), INSERM U964, CNRS UMR7104, Illkirch, France.

[5]Co-last authors

[*]To whom correspondence should be addressed at : EGDM Laboratory, Département Développement, Reproduction et Cancer, Institut Cochin, 24 rue du Faubourg Saint Jacques, 75014 Paris, France. Tel: +33 153732740; Fax: +33 826699430; Email: marco.pontoglio@inserm.fr

Supplementary Figure S1: Absence of *Hnf1a* transcript in both resistant and sensitive mutant strains
Hnf1a expression in P15 pancreas of wild-type 129S2 and CBA, and *Hnf1a*$^{-/-}$ 129S2 and CBA mice was analyzed by RT-qPCR. Number of animals in each group=3; ND=not detected

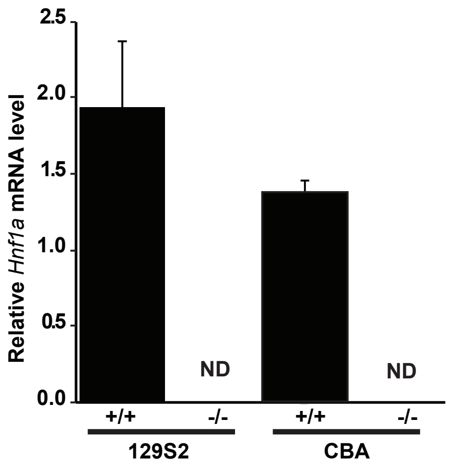

Supplementary Figure S2: No difference in beta cell mass between *Hnf1a* mutant 129B6 and 129CBA mice at P1.5 and P15. Beta cell mass was estimated at **(A)** P1.5 and **(B)** P15 by counting positive cells for insulin labeling and the total tissue area was determined with DAPI staining (3 animals per genotype). Results are expressed as mean ± SD

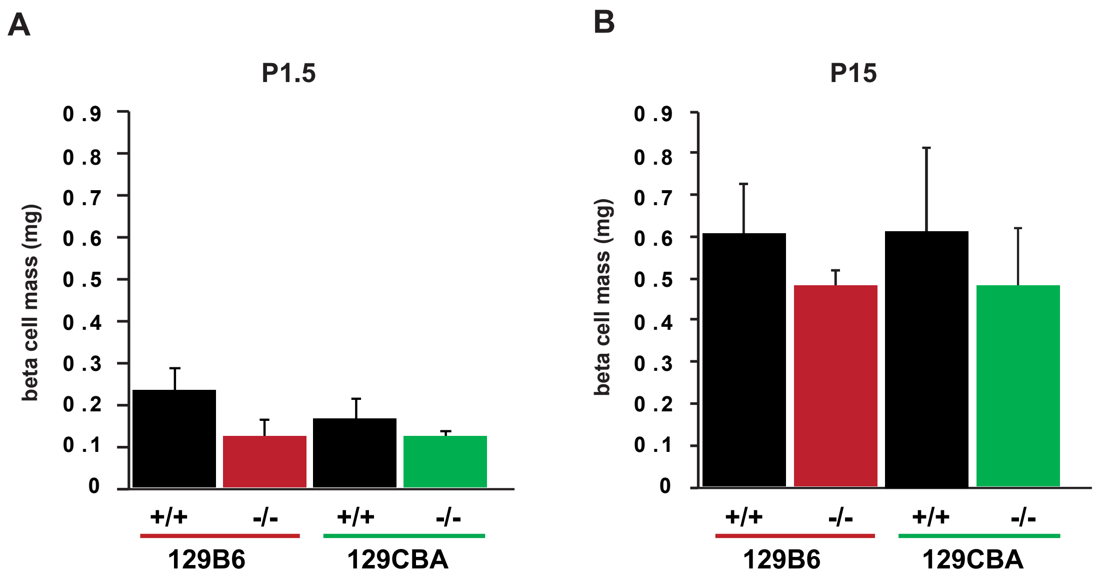

Supplementary Figure S3: Proliferation and apoptosis in beta cells at P15. (A) Proliferation and **(B)** apoptosis were determined by counting the proportion of insulin positive cells labeled with Ki67 antibody or TUNEL assay, respectively**.** No significant differences were observed between the two mutant strains. NS=non-significant; *p-value<0.05. Results are expressed as mean ± SD. Number of animals per genotype=3.

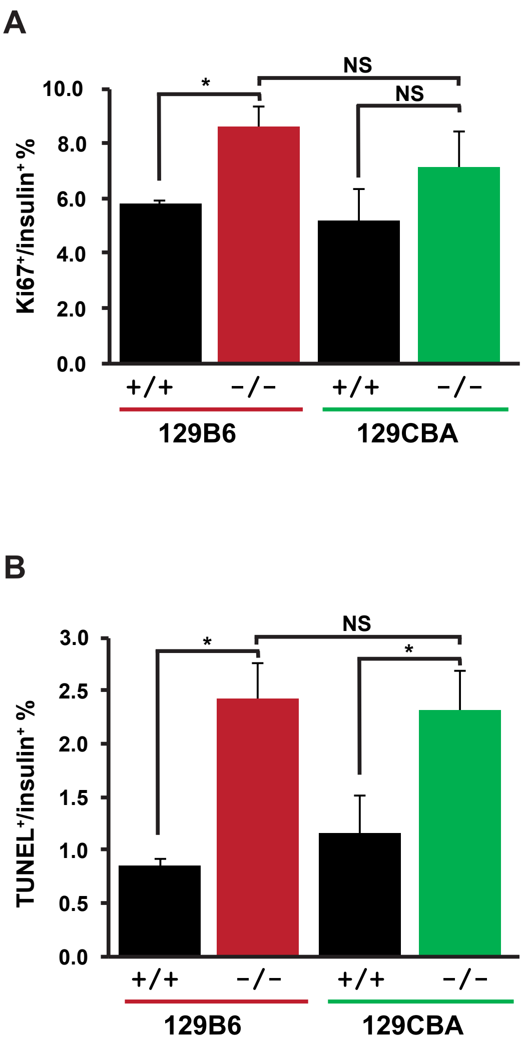

Supplementary Figure S4: Mouse breeding strategy adopted to identify the resistant locus to diabetes. Pure sensitive (129S2) and resistant (CBA) strains heterozygous for Hnf1a mutation were crossed. The resulting F1 progeny was outcrossed with a sensitive strain (B6) distinct from the parental one (129S2) to increase as much as possible the hybrid vigor. N2 Hnf1a null mice derived from this crossing were analyzed for their phenotype.

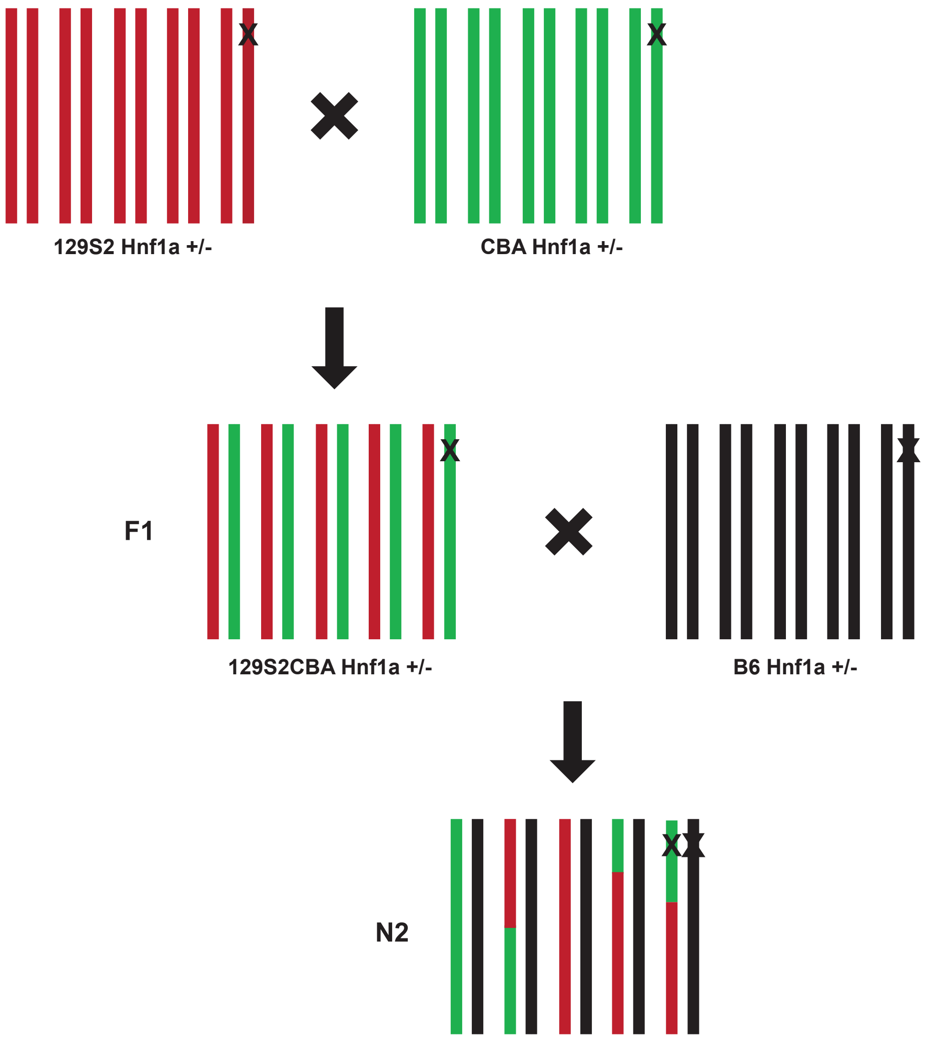

Supplementary Figure S5: Impact of Moda1 allele on the fasting glucose levels in N2 mutant mice. More than 97% of mice that inherited the *Moda1*^{129} allele were hyperglycemic (left part of the chart) whereas 83% of mice carrying the *Moda1*^{CBA} allele did not develop diabetes (right part of the chart). Numbers of mice are indicated in brackets.

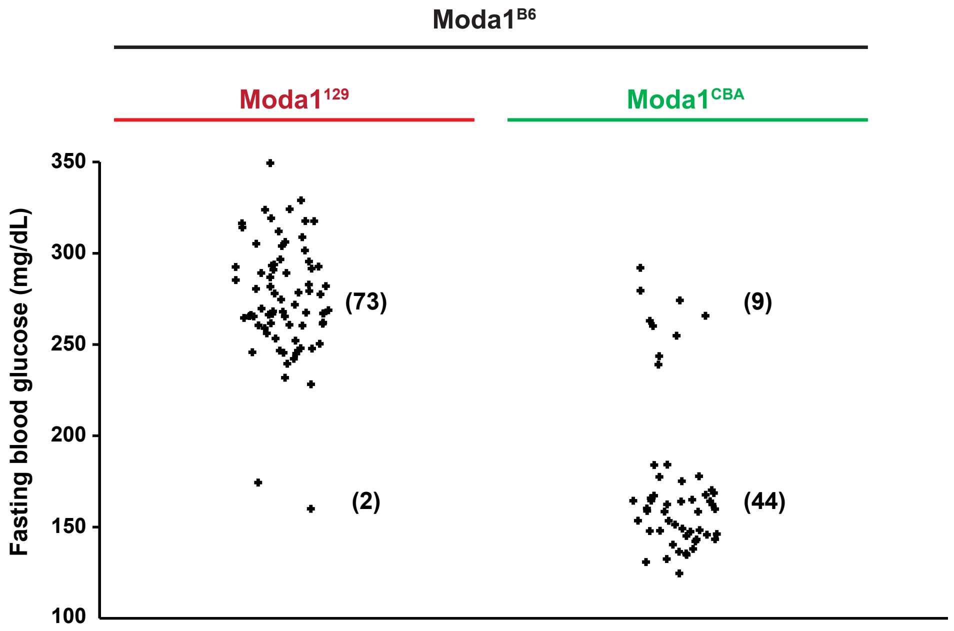

Supplementary Figure S6: Mouse breeding strategy adopted to generate recombinants around the *Moda1* locus. A consomic strain (C3H/HeJ-Chr 3$^{C57BL/6J}$/J or C3H-Chr 3$^{B6/B6)}$) was crossed with a pure C3H strain. The progeny was mated with a C3H *Hnf1a*$^{+/-}$ strain and recombinant mice for the *Moda1* locus were selected. Three independent recombinant lines carrying *Moda1*$^{B6/C3H}$ were then crossed with 129S2 *Hnf1a+/-* strain and mice were challenged with glucose tolerant tests. Long bars denote chromosome 3 and short bars represent the rest of the genome.

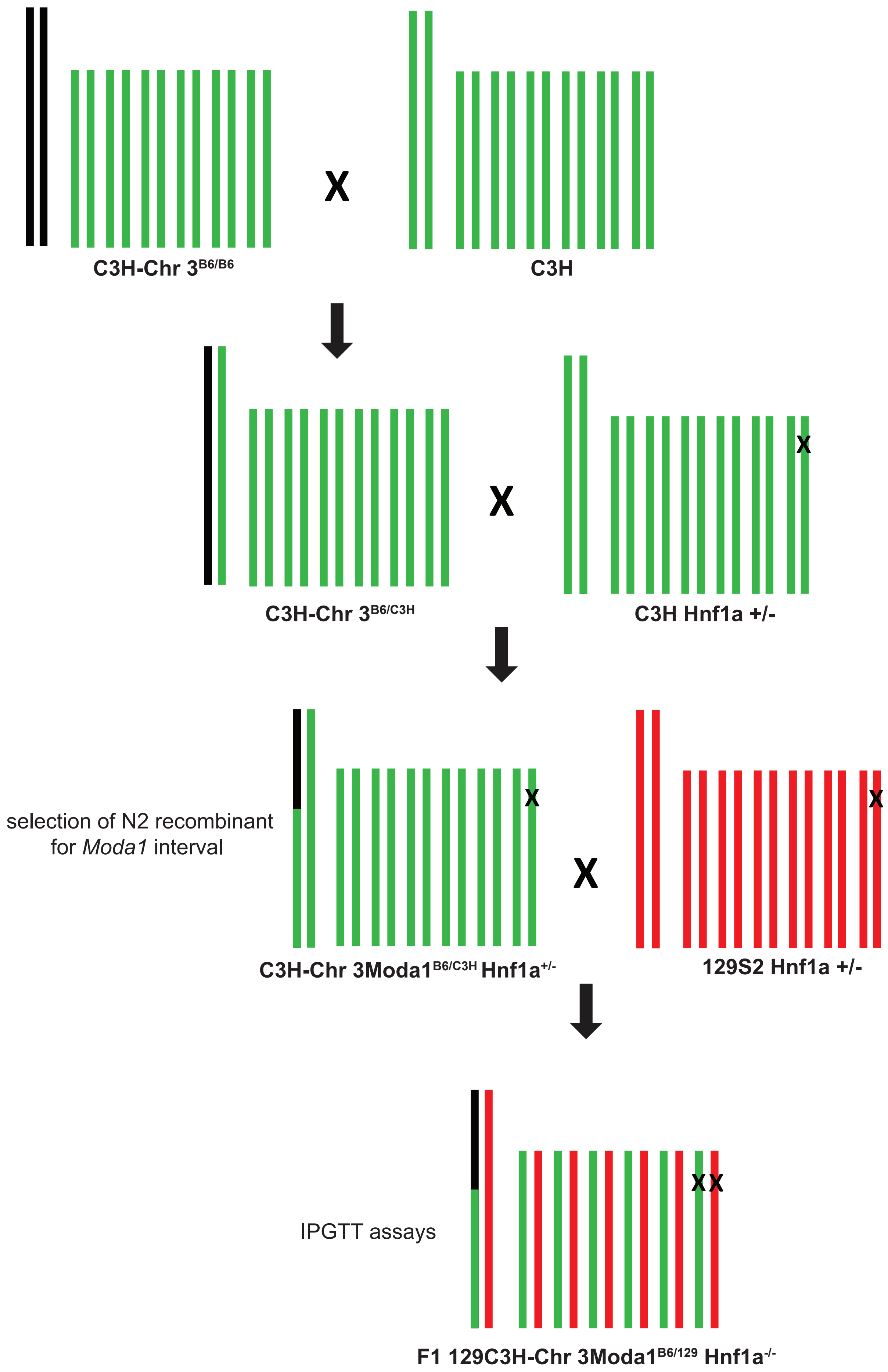

Supplementary Figure S7: The Moda1 interval confers resistance/sensitivity to diabetes

(A) IPGTTs on wild type animals (black curve, n=32), F1 129C3H *Hnf1a*$^{-/-}$ resistant animals (green curve, n=31) and a pool of three distinct recombinant lines that carry *Moda1*$^{B6/129}$ on the chromosome 3 and 129C3H for the rest of the genome (red curve, n=25). Error bars represent SEM. **(B)** Boxplot representation of the area under the curve (AUC) determined from IPGTT presented in **(A)**. AUCs are not significantly different between wild type and F1 129C3H *Hnf1a*$^{-/-}$ mice (black box and green box, respectively). The AUC of recombinant *Moda1*$^{B6/129}$ *Hnf1a*$^{-/-}$ mice (red box) is significantly higher compared to F1 129C3H *Hnf1a* $^{-/-}$ (and wild type). NS=non significant, ***p-value < 0.0001.

A

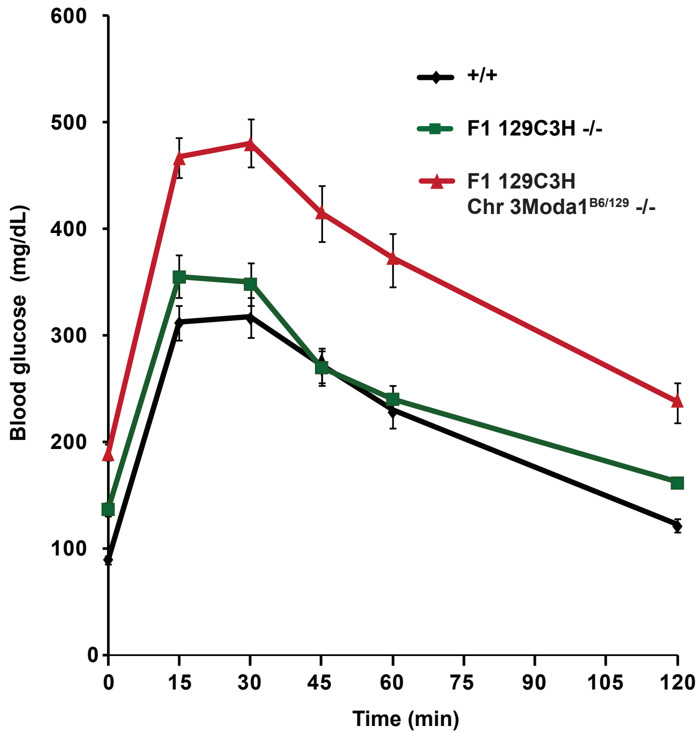

B

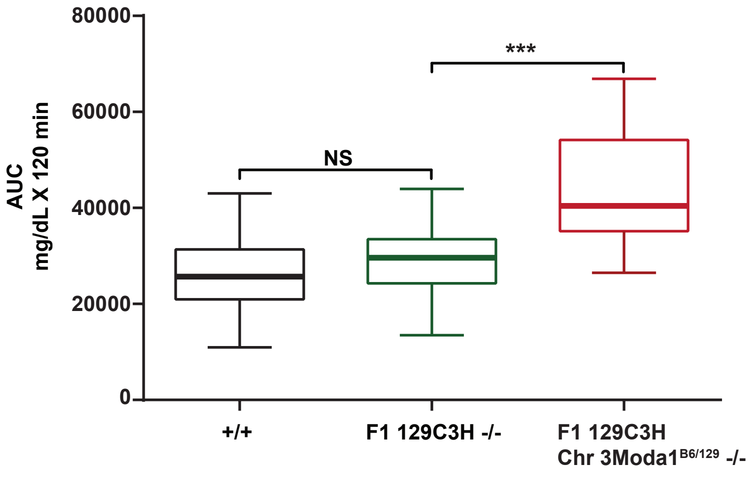

Supplementary Table S1: List of the genes in the *Moda1* locus carrying non-synonymous SNP changes between the CBA/C3H and 129S2 strains. Non-synonymous SNPs, deletions/insertions and structural variants in the QTL interval were obtained from (http://www.sanger.ac.uk/sanger/Mouse_SnpViewer/rel-1303)

Gene symbol	Gene name	Location	db SNP	CBA/C3H allele	Codon change	Amino acid change	Change description
Tmem212	Transmembrane protein 212	27,866,065	rs29598656	T	**C**ac/**T**ac	H/Y	Missense variant
Pld1	Phospholipase D1	28,048,083	rs29602715	G	**C**gg/**G**gg	R/G	Missense variant
Gm1527	Predicted gene 1527	28,895,732	rs50586704	A	c**G**t/c**A**t	R/H	Missense variant
Egfem1	EGF-like and EMI domain containing 1	29,648,265	rs29790718	G	a**C**t/a**G**t	T/S	Missense variant
		29,686,728	rs30410185	C	g**T**t/g**C**t	V/A	Missense variant
Mecom	MDS1 and EVI1 complex locus	30,238,240	rs47702462	C	g**T**t/g**C**t	V/A	Missense variant
Gm10258	Predicted gene 10258	30,268,666	rs29809455	G	**A**tc/**G**tc	I/V	Missense variant
Lrrc31	Leucine rich repeat containing 31	30,689,871	rs37203180	A	**T**ca/**A**ca	S/T	Missense variant
Samd7	Sterile alpha motif domain containing 7	30,756,487	rs31381373	G	**A**ca/**G**ca	T/A	Missense variant
Usp13	Ubiquitin specific peptidase 13	32,902,049	rs29854159	T	a**C**g/a**T**g	T/M	Missense variant
Pex5l	Peroxisomal biogenesis factor 5-like	32,956,647	rs29869735	A	a**G**a/a**A**a	R/K	Missense variant
Ttc14	Tetratricopeptide repeat domain 14	33,800,535	rs49858437	C	**A**tg/**C**tg	M/L	Missense variant
		33,801,070	rs51789388	G	ga**C**/ga**G**	D/E	Missense variant

Supplementary Table S2: Table: Primers used for PCR, RT-qPCR and pyrosequencing

**A. Primers used for PCR genotyping**		
Gene symbol	FORWARD SEQUENCE (5'-3')	REVERSE SEQUENCE (5'-3')
Hnf1alpha	TACCTGATGGTTGGAGAGGGTC	TGAAGACCACATCTCCTAAGG
LacZ	TCAATCCGCCGTTTGTTCCC	GCATAACCACCACGCTCATC
**B. Primers used for PCR Genome scan**		
Position	FORWARD SEQUENCE (5'-3')	REVERSE SEQUENCE (5'-3')
3-del9488256	TCTAACTCCAAACCACTCTGAGG	AGACCCTGGTGTCAAATTGC
3-del13608521	GTTAGCACTGCCCCAAAATG	GCTAACCCATGCTTTCTGGA
D3Mit62	TTCATTTGTTTATGCTTGGGG	TTCATCCTGTCCTCCTCTGG
3-del28725682	TGGCTGACACCTGCATAGAC	CCAGAGATGTCTGCCTGTGA
3-del32716930	CTCGGGACATCGTAAATGCT	GCTATGACACCTGGGTGGAG
3-del35074590	CAGAGATTGAACCCCCTTGA	CTCCTTGAGGCTCTGGATGA
D3Mit224	ATTGCTATACCAAGTAGACAAACACA	ACAAGGAGGCCGCACTAAC
D3Mit189	GTTACCACCCAGAGAAAAGGC	TACTCCTCGCTGCTTCCCTA
D3Mit257	CCTAGCGCAGGAATAGTTAACC	ACAAACAGAACAAAACAAAAAGTCC
D4Mit264	TGAGTCAATAAGGCAGGTTGG	GCAGACCAAACCCCACAC
D4Mit108	TCAGCCATCTCTATCAGGTGG	CCAATGTCAGATAATGCTTATGAG
D4Mit187	AGGTCTTAAGCCCTTTCTCCC	GGAGGGGAATTCAGGAACAT
D4Mit336	TTCATATATGTGTACCATGGCATG	CAGGAACCTACATAGGTGAGAGG
D4Mit158	CCTCATGTGGAGGCCATC	TTCAATTCTCAGAATCCTTGATAGG
D4Mit126	TGCACTTTTGAGATTGCCAG	GTCTTTCCCTCTCCCTCCC
D4Mit226	CCCCCCAAAAAAAAGAGAAA	AGGATTAGTGAAGGCACTGAGG
D11Mit227	CCAGCATTTGAACCCTGATT	AAACCCATAGCCTGCATCTG
D11Mit231	TCTTTAAAATGTTTATGATCTGTGGC	GGCCTTGGATGTGCATAGAT
D11Mit173	AAGTGACATATGGATTCCTGGG	TCAAAGTGGGTATGTGTCATCC
D11Mit242	GAAGCCAGCAAGAAAAATGC	CTGTCTGGTAGTGCAGCCAA
D11Mit301	AATAGTCTCATCGGGTTAAACAGC	AAGTAGACTGATGTGAGGCTAAGTACC
D11Mit214	CATACAGCCTTCAACAATGACA	ACTGCATACATGTGCACTCATG
D18Mit94	TCACCTAGGACCCCCCTC	AAGTAGTGAGAGGCCACCACA
D18Mit206	GCAACCATTCACCATCAGG	TTTTTTTTTTTAAAGACACTAAATGCC
D18Mit153	GCACTTCTGCTTACAAGGCC	CAGGAGTGCAAAGGTCATGA
D18Mit4	ACTGTTGCTGGGGAATGG	CCAAGTTCAAAGCTGCTGG
**C. Primers used to discriminate C3H from B6 allele on chromosome 3**		
Position	FORWARD SEQUENCE (5'-3')	REVERSE SEQUENCE (5'-3')
3-del9488264	CATGCTTGCAAGGACTCTCA	GCTCTATGCTCAGGGGTCAC
3-del26404871	GCAGAAGCAAGCAAACAAAA	ACCGGGCATTCTATGACAAA
3-del27081831	ACGAGCACCCTAAGGACAGA	TCAGCAAGAACAGTGGCATC
3-del28725682	TGGCTGACACCTGCATAGAC	CCAGAGATGTCTGCCTGTGA
3-del30184839	GACACACAGAGTTGGGCTGA	TTGCCATTGCTTTTCCTTTC
3-del35988893	ATCTAGAGACGCACCCCAGA	AGGACAGTGTGCTTGCTCCT
**D. Primers used for RT-qPCR**		
Gene symbol	FORWARD SEQUENCE (5'-3')	REVERSE SEQUENCE (5'-3')
Hprt	AGTCCCAGCGTCGTGATTA	GGAATAAACACTTTTTCCAAATCC
Slc2a2	CGGAATGGTCGCCTCATT	ACATTGCTTTGATCCTTCCAAGTT
Ghsr	CAGACAGTGAAGATGCTTGCT	GGCTCGAAAGACTTGGAAAA
Ghrelin	AGCCCAGCAGAGAAAGGAATC	GGGAGCATTGAACCTGATCTC
**E. Primers used for pyrosequencing**		
Gene symbol	FORWARD SEQUENCE (5'-3')	REVERSE SEQUENCE (5'-3')
Ghsr	TGGTAAAGCAGGCTCAGAATTTC	GAAGAGTGGCATGTGGGTAGA
	SEQUENCING PRIMER	GCATGTGGGTAGATCA
